# Tantalum Nitride-Decorated Titanium with Enhanced Resistance to Microbiologically Induced Corrosion and Mechanical Property for Dental Application

**DOI:** 10.1371/journal.pone.0130774

**Published:** 2015-06-24

**Authors:** Yifei Zhang, Yunfei Zheng, Yongliang Li, Lixin Wang, Yanjie Bai, Qiang Zhao, Xiaoling Xiong, Yan Cheng, Zhihui Tang, Yi Deng, Shicheng Wei

**Affiliations:** 1 Department of Oral and Maxillofacial Surgery, Laboratory of Interdisciplinary Studies, School and Hospital of Stomatology, Peking University Beijing 100081, China; 2 2nd Dental Center, School and Hospital of Stomatology, Peking University Beijing 100081, China; 3 Center for Biomedical Materials and Tissue Engineering, Academy for Advanced Interdisciplinary Studies, Peking University, Beijing 100871, China; 4 Department of Stomatology, Beijing Shijitan Hospital, Capital Medical University Beijing 100038, China; 5 Department of Stomatology, Aviation General Hospital of China Medical University and Beijing Institute of Translational Medicine, Chinese Academy of Science, Beijing 100012, China; North Carolina A&T State University, UNITED STATES

## Abstract

Microbiologically induced corrosion (MIC) of metallic devices/implants in the oral region is one major cause of implant failure and metal allergy in patients. Therefore, it is crucial to develop practical approaches which can effectively prevent MIC for broad clinical applications of these materials. In the present work, tantalum nitride (TaN)-decorated titanium with promoted bio-corrosion and mechanical property was firstly developed *via* depositing TaN layer onto pure Ti using magnetron sputtering. The microstructure and chemical constituent of TaN coatings were characterized, and were found to consist of a hard fcc-TaN outer layer. Besides, the addition of TaN coatings greatly increased the hardness and modulus of pristine Ti from 2.54 ± 0.20 to 29.88 ± 2.59 GPa, and from 107.19 ± 6.98 to 295.46 ± 19.36 GPa, respectively. Potentiodynamic polarization and electrochemical impedance spectroscopy studies indicated that TaN coating exhibited higher MIC resistance in comparison to bare Ti and TiN-coated coating in two bacteria-containing artificial saliva solutions. Moreover, the biofilm experiment showed that the TaN-decorated Ti sample possessed good antibacterial performance. The SEM and XPS results after biofilm removal demonstrated that TaN film remained its integrity and stability, while TiN layer detached from Ti surface in the bio-corrosion tests, demonstrating the anti-MIC behavior and the strong binding property of TaN coating to Ti substrate. Considering all these results, TaN-decorated Ti material exhibits the optimal comprehensive performance and holds great potential as implant material for dental applications.

## Introduction

Oral endosseous implant systems have been developed and used successfully for rehabilitation of partially or completely edentulous patients. However, the high incidence of failures of implant therapy resulting from loss of osseointegration remains unresolved, and medical practitioners will have to face these challenges in their daily practice. Subgingival biofilm has been proved to be a well-established etiologic factor and cause for loss of marginal bone and implant failure [1]. Moreover, long term presence of bio-corrosion reaction contributs to the accumulation of bacteria and leads to fractures of the implants. Hence, implant materials with high biocompatibility and optimal quality of minimizing the accumulation of peri-implant plaque have drawn considerable attention [2]. Currently, titanium (Ti) and Ti-based alloys are widely used for oral endosseous materials such as implants and prosthetic crowns because of their biocompatibility, biostability, and unique mechanical properties [3,4]. Ti materials, nevertheless, exhibit insufficient abrasion/corrosion resistance and inferior antimicrobial property in some oral circumstances containing fluoride ions, lactic acid and microorganisms [5–8]. Moreover, allergy to Ti due to particles of eluted Ti in the human body and discoloration of Ti are becoming a concern when Ti is used as a dental material [9,10].

The word microbiologically induced corrosion (MIC) or bio-corrosion is defined when the participation of bacterial activity exacerbates the corrosion cycle of metal devices [11,12]. An inter-relationship exists between oral microorganisms and dental biomedical materials applied *in vivo*: dental materials exert influence against the adhering biofilms, whereas microorganisms accelerate the corrosion of metal and alloys immersed in an aqueous environment [13]. The long-term presence of bio-corrosion would further contribute to compromised mechanical properties, even fractures of the alloy-abutment interfaces, abutments, and implant bodies [14]. Therefore, the surface modification of Ti surface by coatings to provide additional protection and antimicrobial behavior has become the subject of extensive studies and is necessary to improve clinical treatments.

Over the past two decades, significant strides been made in developing improved biointerfaces of Ti implant. Various anti-bioactivities, biocompatibility-enhancing properties, and corrosion resistance through thin-film coating including layer-by-layer technology [15,16], polyelectrolyte multilayer [17], and electrophoretic strategy [18] have been reported. For instance, the controlled-release antibacterial coatings loaded with minocycline were constructed *via* self-assembly of chitosan and alginate using the layer-by-layer technique on Ti substrate [16]. Peterson et al. reported that a polyelectrolyte multilayer coating with adsorbed growth factors (BMP-2 or FGFb) based on poly(methacrylic acid) and poly-L-histidine formed on Ti surfaces showed a great promotion on cell proliferation and differentiation [17]. On the other hand, the chemical stability and mechanical properties of Ti can be enhanced through surface nitridation and oxidation making the surface free from unsaturated bonds for further interactions. So coating with titanium nitride (TiN) [19] or zirconia (ZrO_2_) [20] is frequently recommended to improve the shear strength and corrosion resistance for implant abutments made of Ti. Although TiN is relatively stable, it will be oxidized upon exposure to oxygen or air foming TiO_2_ layer [21], whose integrity and stability also might be weaken by metabolites produced from microorganisms. Studies also reveal that increased number of bacteria is evident on TiN film compared with the pristine Ti, and TiN coating shows osseointegration similar or worse to that of Ti [22,23]. Similarly, coating with ZrO_2_ demonstrated limits potential to reduce the adhesion of bacteria [24], hampering their applications in clinic. Thus, the search for new coating material which resists the adhesion of bacteria should be encouraged.

Tantalum (Ta) has become a promising metal for biomedical implants or implant coatings for the orthopedic, dental and cardiovascular applications since the 1940s owing to its excellent corrosion resistance, radio-opacity, biocompatibility, and hemocompatibility [25–27]. Similar to Ti, Ta is highly unreactive and biocompatible in the body. Previous literature pronounces that Ta element is good for the osteogenesis in animal implantation tests, and favourable for cell adhesion, proliferation and differentiation *in vitro* studies [27,28]. Among Ta compounds, tantalum nitride (TaN), which has been widely used in surface modification of mechanical tools, possesses better corrosion resistance, stable chemical properties [29], and its hardness is higher than that of TiN [30]. Recent studies show that Ta-based coatings including TaN and tantalum oxide (TaO) coating exert antibacterial effect against *Staphylococcus aureus* and *Actinobacillus actinomycetemcomitans* [31,32]. Despite the excellent biocompatibility/cytocompatibility of TaN has allowed its use in artificial heart valves [33], dental implant [32] and other numerous biomedical coatings [30,32], e.g. a Ag-doped TaN film with suppressed bacterial activity was developed by Huang et al. on Ti-based dental implants [32], the impact of bio-corrosion resulting from bacteria on the pristine TaN-decorated implant abutment in the oral cavity, to our knowledge, has not been reported and investigated. Hence, in our study, a pilot and preliminary work was conducted on the fabrication of TaN coatings onto Ti substrate by magnetron sputtering, and the anti-MIC in bacteria-containing artificial saliva (AS) media and biofim formation were also assessed. Compared with the past thin coatings deposited on Ti, this novel TaN coating could effectively enhance its mechanical property, prevent the MIC and increase the antibacterial performance of Ti, presenting a bona fide potential as implant or coating material for dental application.

## Materials and Methods

### 2.1. Sample preparation

Prior to surface modification, Ti substrates (commercial pure Ti, grade 2, purchased from Northwest Institute for Non-ferrous Metal Research, Xi’an, China) were cut into discs of 15 mm in diameter and 2 mm thick. These substrates were polished with a series of SiC abrasive papers (400, 1000, 1500, and 2000 grit) to a mirror-finish surface, cleaned ultrasonically for 20 min in baths of acetone, anhydrous ethanol and de-ionized water (D.I. water), respectively, and then dried at 60°C overnight. The Ti substrates were then coated with tantalum nitride (TaN-coated Ti) as specified below, and titanium nitride-coated Ti (TiN-coated Ti) and polished Ti specimens (uncoated Ti) were used as controls.

Briefly, TaN and TiN coatings were deposited using asymmetric bipolar pulsed reactive magnetron sputtering in a multi-functional coating rig (TSU-650). The sputtering targets were pure Ta (99.99% purity) and Ti (99.99% purity) for the deposition of TiN and TaN thin films. Before deposition, the substrate surfaces were cleaned with argon (Ar, 99.99% purity) for 15 min at a working pressure of approximately 1.4 Pa and a negative substrate bias of -800 V. The sputtering process has been performed at ambient temperature with the target diameter of 76.2 mm. The distance from the magnetron to the sample was 13 cm, and the tilt of magnetron to the substrate normal was set to 35°. During the sputtering process, the substrate holder rotated at a speed of 12 rotations per minute and a mixture of Ar and N_2_ gas (99.99% purity) was used for the reactive formation of nitride films. For TaN deposition, Ar/N_2_ gas was mixed into the chamber at a working pressure of 0.3 Pa with a flow ratio of 30:3.5, and the source power of the Ta targets was 120 W. For TiN deposition, the total Ar/N_2_ gas pressure was 0.5 Pa, with a flow ratio of 30:1.2 and the source power of the Ti targets was 150 W. The procedure for TaN/TiN deposions was lasted for about 180 min, and the resulting products were cleaned with anhydrous ethanol and D.I. water, respectively before use and test.

### 2.2. Surface Characterization

The phase composition of the prepared TiN- and TaN-decorated Ti was examined and compared by X-ray diffraction (XRD, Shimadzu, Japan) using a Cu target as radiation source (λ = 1.540598 Å) at 40 kV. The diffraction angles (2θ) were set between 25° and 90°, with an incremental step size of 4° min^-1^. Identification of phases was achieved by comparing the obtained sample diffraction pattern with standard cards in the ICDD-JCPDS database.

The thickness of the TiN and TaN films was measured by a Veeco Dektak 150 profilometer (USA), with a measurement error about plus or minus 5 nm.

X-ray photoelectron spectroscopy (XPS, AXIS Ultra, Kratos Analytical Ltd., Japan) was employed to identify the chemical constituents and elemental states of the different coated Ti samples. The binding energies were calibrated by the C 1s hydrocarbon peak at about 285 eV. The quantitative analysis and the curve fitting were conducted by the CasaXPS software package.

Contact angles on the pristine and decorated Ti were measured at room temperature by the sessile drop method using 2 μL D.I. water droplets in a contact angle measuring device (SL200B, Kono, USA). Six samples in each stage were used to provide an average and standard deviation.

The morphology of the unmodified and modified Ti substrates was characterized by using a field emission scanning electron microscope (FE-SEM, JSM-6701F, JEOL, Tokyo, Japan). All samples were coated by gold for 1 min before SEM observation.

### 2.3. Mechanical properties and adhesion strength tests

The elastic modulus and hardness of studied materials were determined by a nanoindentation test using an *in situ* nanomechanical test system (Hysitron, USA) equipped with a diamond Berkovich pyramidal tip (50 nm) at an angle of 65.3° between the tip axis and the faces of the triangular pyramid. Prior to measurement, the indenter was calibrated by measuring the elastic modulus of a fused silica standard sample (69.9 GPa). The instrument was operated in continuous stiffness mode (CMS) and measurements were made within an indentation depth of 1 μm. Ten indentations were made on each sample. Simultaneously, the morphological characteristics and surface roughness of TiN- and TaN-coated Ti were assessed *via* the equipped *in situ* scanning probe microscope (SPM). The scan range was 10 μm × 10 μm, and scan rate was 0.5 Hz. Before nanomechanical measurement, the different Ti substrates were rinsed with ethanol and D.I. water, and allowed to air dry.

The adhesion force between the coating films and Ti substrate was evaluated employing an Auto Scratch Coating Tester per previous descriptions [30]. The scratch test consisted of the generation of scratches using a spherical stylus (Rockwell C diamond) maintained at a constant speed over the surface under different test loads. The critical load was defined as the smallest load at which a recognizable failure occurs. The failure force was determined from the load versus acoustic output characteristics.

### 2.4. Bacteria strains and culture

*Streptococcus mutans* (*S*. *mutans*, UA159 obtained from American Type Culture Collection, USA) was inoculated on Brain Heart Infusion (BHI, Becton Dickinson, USA) plates, and incubated under microaerophilic conditions (5% CO_2_) at 37°C. *Actinomyces viscosus* (*A*. *viscosus*, ATCC19246 obtained from American Type Cell Collection) and *Porphyromonas gingivalis* (*P*. *gingivalis*, ATCC 33277 obtained from American Type Culture Collection, USA) were revived on CDC blood plates, consisted of 15 g/L tryptone, 5 g/L soya peptone, 5 g/L yeast extract, 5 g/L sodium chloride, and 0.4 g/L L-cysteine, 1 mg/mL menadione, 5 mg/mL haemin, and 25 mL of sterilized defibrinated sheep’s blood, and grown under anaerobic conditions at 37°C. After 48 h culture, these bacteria were selected and cultured in a modified BHI medium. Each liter of the medium consists of 37 g BHI, 5 g yeast extract and 1g L-cysteine. The pH was adjusted to 7.4 ± 0.1 using a 2 M NaOH solution and sterilized by autoclaving for 20 min at 121°C and at 0.2 MPa. The bacteria growth was estimated by absorbance at 630 nm wavelength using a microplate reader (Elx808, Bio-tek, USA), and the optical density was about 0.3–0.4 for each bacteria fluid.

### 2.5. Electrochemical evaluations in bacteria-containing artificial saliva

#### 2.5.1. Preparation of bacteria-containing artificial saliva

The Fusayama-Meyer AS was prepared as described previously [34] containing 0.906 g/L CaCl_2_·2H_2_O, 0.690 g/L NaH_2_PO_4_·2H_2_O, 0.4 g/L KCl, 0.4 g/L NaCl, 0.005 g/L Na_2_S·9H_2_O, 1 g/L urea and 5 g/L yeast extract. In order to better understand MIC in oral environment, two bacteria-containing AS solutions were prepared: 1) *S*. *mutans* liquid after enriched cultivation was added to AS medium at the ratio of 1:50 denoted as AS-*S*.*mu*; 2) *A*. *viscosus* liquid after enriched cultivation was added to AS medium at the ratio of 1:50 named as AS-*A*.*vi*.

#### 2.5.2. Electrochemical experiment

The anti-corrosive activities of uncoated Ti, TiN-coated and TaN-coated Ti against *S*. *mutans* and *A*. *viscosus* were evaluated by open circuit potential (OCP)-time measurements, electrochemical impedance analysis, and anodic polarization experiments. The electrochemical properties of the studied material surfaces were measured in a glass electrochemical cell at 37 ± 0.1°C which was connected to a computer-controlled potentiostat (CHI 650C, Chenhua, Shanghai, China). Corrosion tests were performed using the standard 3-electrode cell method [saturated HgCl as the reference electrode, Pt as the counter-electrode, and the exposed surface (1.77 cm^2^) of the decorated Ti samples as the working electrode]. The OCP of each specimen was continuously monitored for 2 h in electrolytes. Electrochemical impedance spectroscopy (EIS) at the open-circuit potential was carried out with an AC amplitude range of -10 mV to 10 mV and evaluated in the frequency range from 10^−2^ Hz to 10^4^ Hz. The EIS data were interpreted in Bode amplitude and phase angle plots using ZSimpWin program. After EIS test, the potentiodynamic polarization measurement was performed with a scan rate of 1 mV·s^-1^. Corrosion parameters including corrosion potential (*E*_*corr*_) and corrosion current density (*I*_*corr*_) can be estimated from the polarization curves by Tafel analysis based on the polarization plots.

### 2.6. Biofilm formation in mixed bacteria solutions

#### 2.6.1. Incubation of samples in mixed bacteria solutions

The different Ti samples placed in 24-well plates were first immersed in sterile AS for 4 h at 37°C in an incubator. Afterwards, they were incubated for periods of 14 days in 2 mL of modified BHI medium containing 30 μL of *S*.*mutans* (OD_630_ = 0.25), *A*.*viscosus* (OD_630_ = 0.39) and *P*. *gingivalis* (OD_630_ = 0.27) at 37°C under anaerobic condition to allow the biofilm formation. To maintain the culture nutrition, the medium was renewed every 48 h.

#### 2.6.2. Biofilm formation observation

Biofilm formation on the surface of pure Ti, TiN-coated and TaN-coated Ti samples was probed using SEM, white light interferometer (WLI), and confocal laser scanning microscope (CLSM) after 14 days culture. Samples were fixed in 2.5% glutaraldehyde solution in PBS for 30 min and then dehydrated with a series of graded ethanol solutions (50, 75, 80, 95, 100%) for SEM observation. Dehydrated samples were dried by a vacuum dryer before sputter-coating with gold using a sputter coater. A WLI (Mapvue AE Version 2.24, KLA-Tencor, USA) was used to capture the images of biofilms on the coupon surfaces with vertical scan range of 30 μm and the scan rate of 2.1 μm/s. A LIVE/DEAD BacLight bacterial viability kit (L-7007, Invitrogen, USA) was used to determine the long-term bacterial cell viability on the surfaces for CLSM. In this assay, the red-fluorescent nucleic acid staining agent propidium iodide, which penetrates only damaged cellmembranes, was used to label dead bacterial cells. By contrast, the SYTO9 green-fluorescent nucleic acid staining agent, which can penetrate cells with both intact and damaged membranes, was used to label all the bacterial cells. After 14 days incubation, the supernatant was removed, and the substrates were washed with PBS buffer at least three times. They were then incubated with 400 ml of a dye-containing solution, which was prepared by adding 6 ml of SYTO (3.34 mM) and 6 ml of propidiumiodide (20 mM) to 4 ml of PBS buffer at room temperature in the dark for 15 min, per the manufacturer’s protocol. The stained bacterial cells were examined under a Zeiss LSM510 laser scanning confocal microscope (Germany).

### 2.7. Morphological and chemical characterization of corrosive surfaces

#### 2.7.1. SEM and in situ SPM observation

After 7 days and 28 days of exposure to the mixed bacteria, the bare Ti, TiN-coated and TaN-coated Ti coupons were retrieved from the inoculated medium. The biofilm/bacteria were cleaned sequentially in D.I. water and ethanol, respectively, for approximately 5 min in an ultrasonic bath. In addition, the coupons were exposed to the pure M-BHI medium without bacteria to act as the corresponding control groups. These treated samples were sputter-coated with Au and analyzed by FESEM (JEOL, Japan) at 20 kV and In situ SPM (Hysitron, USA) for observation of the surface morphology on the decorated Ti after MIC.

#### 2.7.2. XPS analysis of corrosive surfaces

XPS was used to determine these corrosive surface elemental components and their chemical states. XPS was performed (AXIS Ultra, Japan) using a monochromatic Al Ka electrode at 15 kV and 150 W at a 45° take-off angle. The binding energies were also calibrated by the C 1s hydrocarbon peak at about 285 eV. The quantitative analysis was conducted by the CasaXPS software package, and the high-resolution spectra of Ti 2p, Ta 4f, O 1s and N 1s were de-convoluted for the pure Ti, TiN-coated and TaN-coated Ti samples, respectively.

### 2.8. Statistical analysis

All data are presented as the mean ± standard deviation (if not mentioned). Statistical analysis was performed with Origin software. Student’s *t*-test was used to determine the significant differences among the groups, and *p*-values less than 0.05 were considered statistically significant.

## Results and Discussion

### 3.1. Microstructure of TiN and TaN coatings

**Fig 1:**
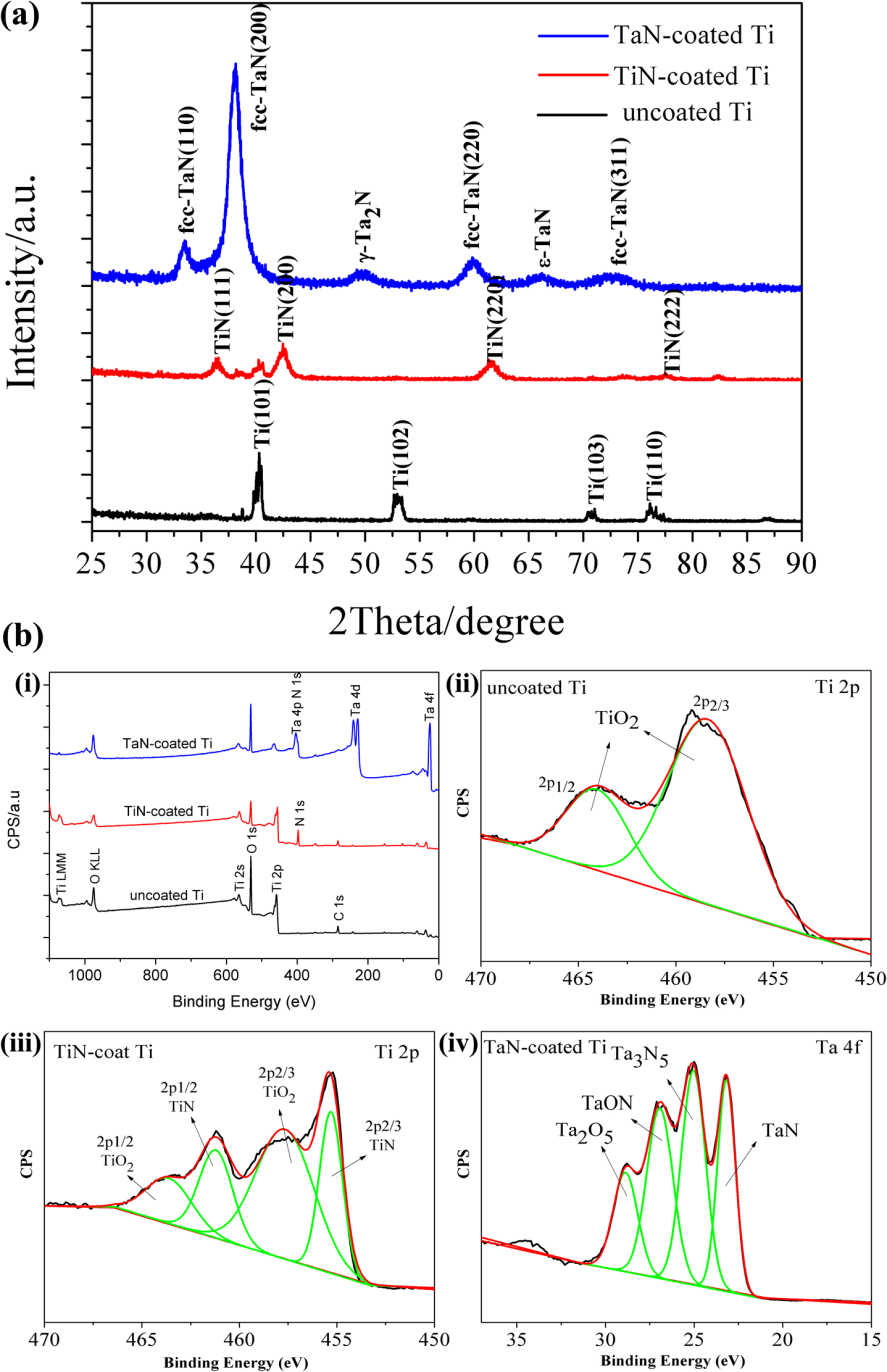
XRD patterns (a) and XPS survey scan spectra (b) of the pristine Ti, TiN-coated and TaN-coated Ti: (bi) XPS wide spectra; the high-resolution Ti 2p spectra for the pristine Ti (bii), TiN-coated Ti (biii), and Ta 4f spectrum for TaN-coated Ti (biv).

Fig 1(A) showed the typical glancing angle XRD patterns of the resulting coated Ti substrates. The Bragg diffraction peaks of the pristine Ti at 2θ values of 40.8°, 53.1°, 71.3° and 76.4°, indexed to (101), (102), (103) and (110) planes respectively, were consistent with the peaks of pure tatinum (PDF #65–3362). In accordance with previous studies [35], the peaks of TiN-coated Ti samples were attributable to the (111), (200), (220) and (222) planes of TiN, and the (200) crystalline plane was dominant in the TiN layer indicating a single-phase NaCl-type cubic structure. However, we identified a mixed face centered cubic (fcc) δ-TaN [PDF #49–1283], hexagonal ε-TaN [PDF #39–1485] and hexagonal γ-Ta_2_N [PDF #26–0985] phases in the XRD patterns of the as-deposited TaN coating on Ti. It is reported that Ta-N is a complex system with more than 11 reported equilibrium and metastable phases depending on the different deposition conditions such as the flow rate ratio Ar/N_2_ and annealing temperation [36]. Also, the dominant phase varies from tetragonal β-Ta phase, prepared in pure Ar, to cubic TaN_0.1_, to hexagonal Ta_2_N, to face centered cubic TaN with increasing the nitrogen flow ratio in the discharge [37,38]. In the persent study, the face centered cubic δ-TaN identified from the (111), (200), (220) and (311) reflections was found to be the major phase compared to hexagonal phase. Next the thickness of coating films was tested by a Veeco profilometer because thickness would be an important parameter to clarify the following analysis. The thickness of the TiN film deposited on the Ti substrate was 727 ± 31 nm, while the TaN layer was 1026 ± 31nm, thicker than TiN coating.

### 3.2. XPS analysis of TiN and TaN coatings

To verify the results from XRD, XPS, a surface-sensitive characterization technique, was applied to evaluate the detail chemical bonds formed on the surfaces of the TiN- and TaN-functionalized Ti surfaces. The XPS survey spectra and quantitative XPS analysis results were shown in Fig 1(B) and Table A in S1 File. The uncoated Ti showed strong Ti 2p, Ti 2p- and O 1s peaks as well as C 1s (Fig 1(Bi)). Carbon, typically present from unavoidable hydrocarbon contamination, was used as an internal reference at 284.6 eV for calibrating peak positions [39]. Whereas nitrogen peaks newly appeared in the XPS spectra and the Ti/N ratio was about 2.15:1 for the TiN-decorated Ti sample. Successful deposition of TaN on the Ti substrate was indicated by an appearance and increase in Ta and N contents. Furthermore, complete suppression of photoelectron peaks unique to Ti 2p confirmed formation of the TaN thin film. The stoichiometry of Ta/N ratio was estimated based on the Ta 4f and N 1s spectra data, and the Ta/N ratio in TaN coating was about 2.24:1, higher than Ti/N ratio in the TiN layer. Moreover, an evident change in Ti and Ta bond composition observed in the high-resolution narrow spectra clearly supported these conclusions (Fig 1(Bii–Biv)). The chemical states of Ti 2p on the surface of uncoated Ti were composed of spin-orbit splitted doublet Ti 2p3/2 at 458.5 eV and Ti 2p1/2 at 465.2 eV, respectively, indicating that TiO_2_ was a main component in the surface layer of Ti samples [40]. The high-resolution Ti 2p spectrum of the TiN-coated Ti, neverthless, was deconvoluted into four curves. Beside oxidative state of Ti 2p2/3 and Ti 2p1/2, the binding energies centred around 455.1 and 461.6.2 eV were assigned to the Ti-N bonds [41], suggesting the presence of TiN. As for TaN-coated Ti samples, oxidative and nitrided states were obvious: the high-energy-side doublet (Ta 4f = 26.1 eV and 28.2 eV) of the Ta spectrum was in agreement with the Ta-O phases (TaON and Ta_2_O_5_) [42]. The O element ineluctably incorporates into the surface because Ta has high oxygen affinity and is easy to suffer from oxidation when exposure to oxygen. While the binding energy values of the low-energy-side doublet (Ta 4f = 23.9 eV and 25.3 eV) should be attributed to Ta-N phase (TaN and Ta_3_N_5_) [42], implying sucessful coating of TaN layer on the surface of Ti.

Additionally, in the high-resolution N 1s spectra of TiN-coated Ti surface (Figure A in S1 File), the peaks at about 398.6 eV and 397.5 eV, related to the-O-NH- and N-Ti bond, were found. In the spectrum of TaN-decorated Ti surface (Figure A(c) in S1 File), XPS signal of N 1 s was observed at binding energy at around 397.1 eV (N 1s) and Ta 4p3/2 was detected at binding energy at around 404.9 eV, which were in good agreement with previous works [43], further proving the successful TaN coating. De-convolution of O 1s core level peaks revealed the existence of TiO_2_, Ta_2_O_5_ and hydroxyl groups on the decorated Ti surfaces. Figure A(b) in S1 File showed the de-convoluted O 1s shifts contained two peaks, one peak at 532.8 eV that belonged to hydroxyl bond and second peak at 530.7 eV corresponding to Ti-O groups from TiO_2_ phase. Similarly, two peaks were seen in TaN-coated Ti sample, and peak at approximate 531.7 eV was attributed to oxides of tantalum (Ta_2_O_5_). These results obviously suggested that TiN and TaN coating were immobilized onto the pristine Ti surfaces.

### 3.3. Surface morphology and roughness

**Fig 2:**
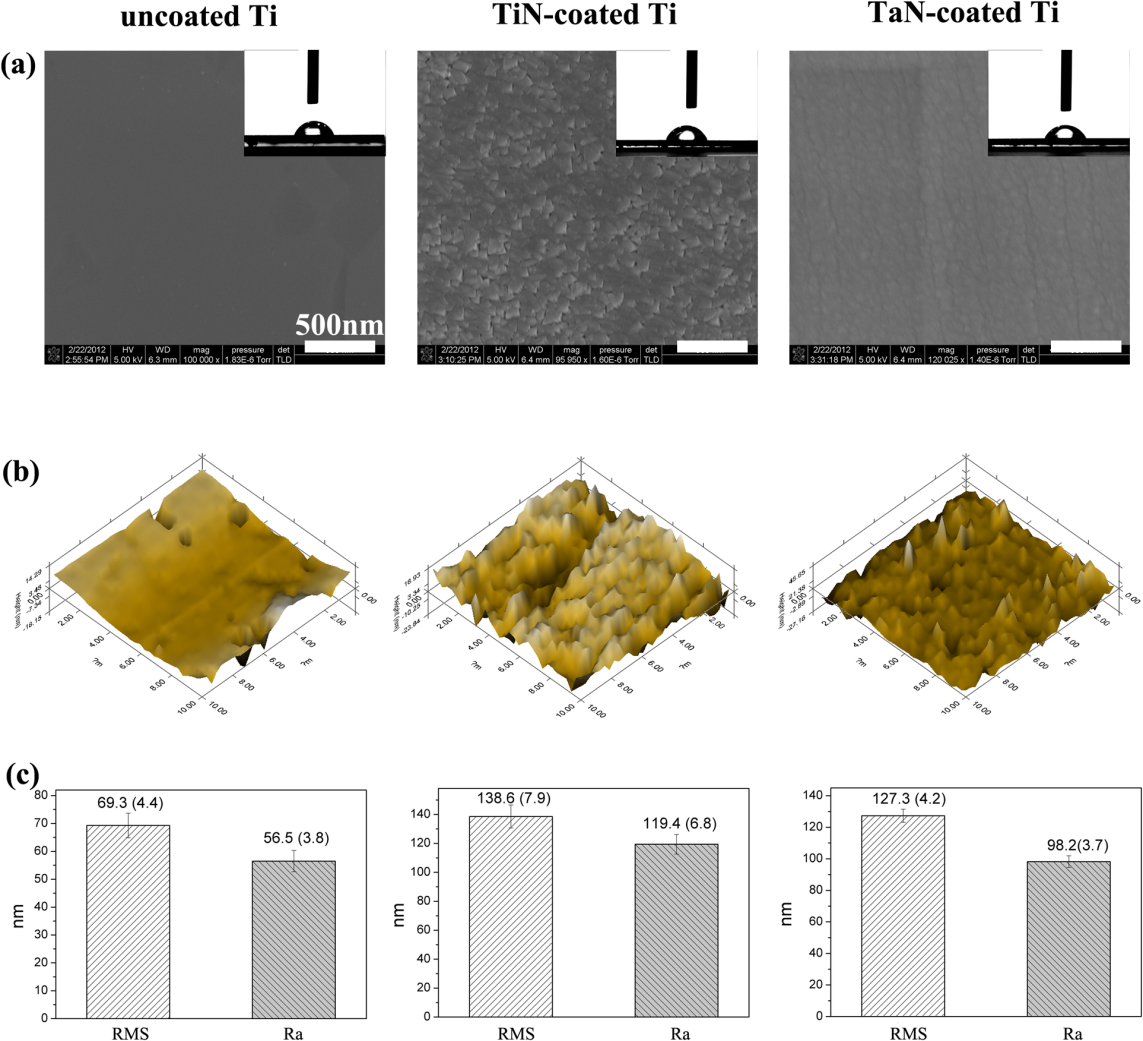
Surface characteristics of the pristine Ti, TiN-coated Ti and TaN-coated Ti samples: (a) SEM images, the insets show the water contact angle of the corresponding materials; (b) in situ SPM images; and (c) surface roughness, root-mean-square roughness (RMS) and average roughness (Ra) are used to present the surface roughness.

Following chemical characterization, SEM and *in situ* SPM were performed to measure the surface topographies, since surface morphology is one of the most pivotal factors in determining the biocompatibility and microbial compatibility of biomedical materials. As depicted in Fig 2(A), SEM investigations revealed the dramatic differences in the surface morphology between Ti, TiN-coated Ti and TaN-coated Ti. The neat Ti had a flat morphology and no obvious pores and coating layer were observed. Although, many nanoscale protuberances were evident on the surface of the TiN-coated and TaN-coated Ti samples, both decorated Ti samples exhibited a good uniform and compact surface. SPM image analysis was carried out for further investigation of the surface morphology and roughness. It was observed that bare Ti surface was smooth as shown in Fig 2(B), however, after addition with TiN and TaN layers, the surface morphologies of coated Ti became rougher than pristine Ti, coincided with the SEM images. Root-mean-square roughness (RMS) and average roughness (Ra) were employed to present the surface roughness. It was found that the uncoated Ti had a low surface roughness of 69.3 ± 4.4 nm in RMS and 56.5 ± 3.8 nm in Ra, while about two-fold increase in the surface roughness was witnessed when the Ti was sputtered with TiN and TaN coatings. On the other hand, it is recognized that the surface hydrophilicity of biomaterials is crucial to their biofuncitons. Hence, the hydrophilicity and surface energy of the samples was evaluated by the static sessile drop on the various Ti samples reflected in the top-right insets in Fig 2(A) and Table B in S1 File. The modification with TaN and TiN coatings led to no significant alteration in the contact angles and surface energy of Ti, as all the water contact angles (CA_water_) closed to 76° and surface energy was about 41 J/m, indicating of similar hydrophilic properties.

### 3.4. Mechanical properties of TiN and TaN coating

**Fig 3:**
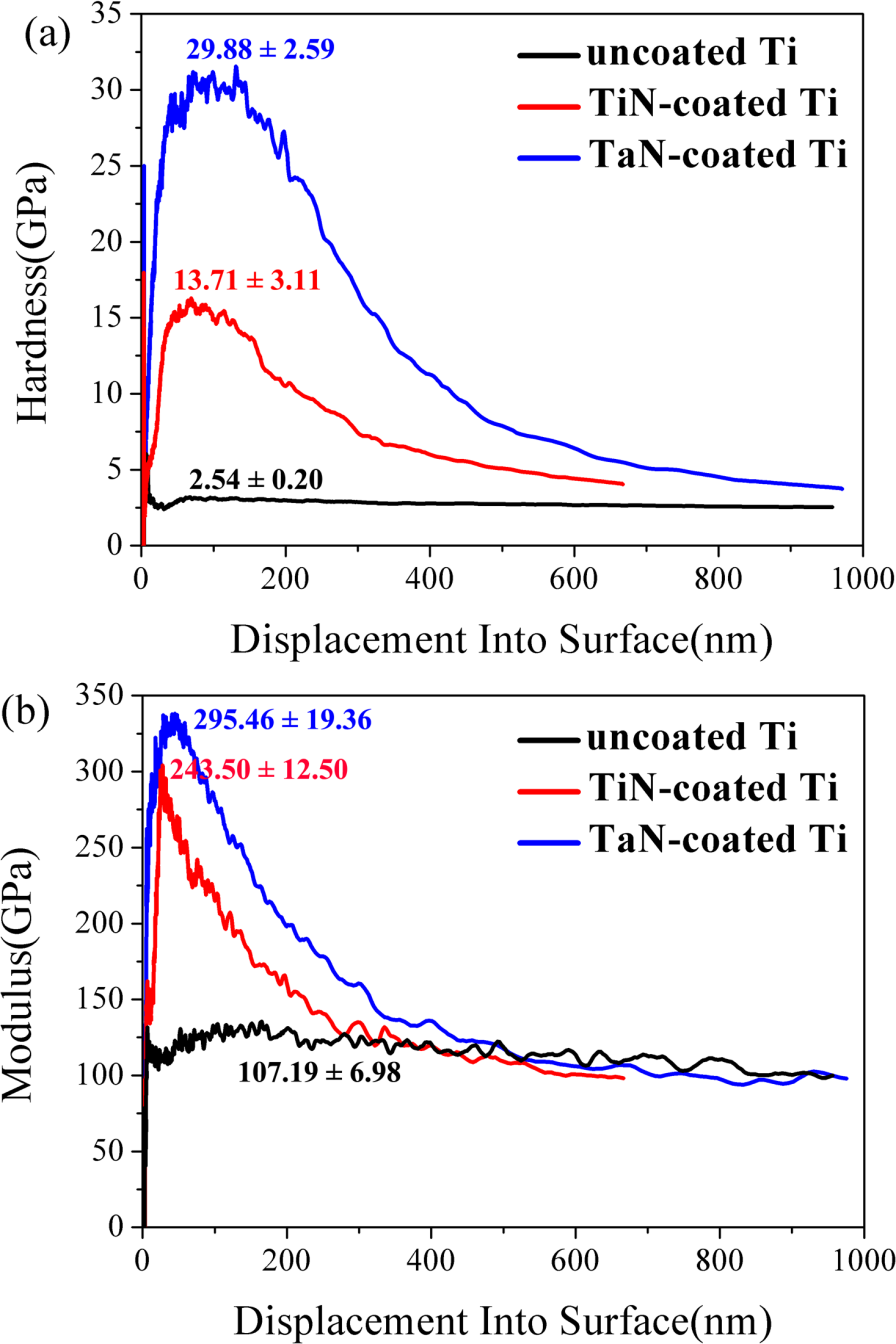
Hardness (a) and elastic modulus (b) *vs*. depth curves for the uncoated Ti, TiN-coated Ti, and TaN-coated Ti samples.

It is well known that artifical dental implant should meet the requirement of high mechanical properties, and scratches are easily generated on the surface of pure Ti metals when they are used as dental implant. Hence, there is an urgent need to improve the mechanical properties of Ti. The typical indentation hardness-depth and modulus-depth curves of the prepared pure Ti, TiN-coated and TaN-coated Ti were shown in Fig 3. Although the surface morphology could influence the hardness testing results [44,45], TiN-coated Ti sample (119.4 ± 6.8 nm) had similar surface roughness to TaN-coated sample (98.2 ± 3.7 nm), resulting in the similar effect on the hardness results, hence, the differences in hardeness values between groups arised from distinct coatings on Ti surface. Due to the reinforcing effect of TiN layer on the Ti surface, the hardness (H) and elastic modulus (E) of Ti both increased from 2.54 ± 0.20 to 13.71 ± 3.11 GPa and from 107.19 ± 6.98 to 243.50 ± 12.50 GPa, respectively. Compared with TiN-coated Ti, TaN-coated Ti displayed higher hardness (29.88 ± 2.59 GPa) and elastic modulus (295.46 ± 19.36 GPa) as a result of the excllent mechanical properties of TaN layers. Besides, the adhesion strength between the film and substrate is also a very important factor for good mechanical properties of devices/implants in the oral region. The results obtained by scratch test showed that the adhesion strength of TaN was about 26.7 N, more than two times of pure TiN coating with the value of approximately 12.1 N. From earlier literature, the TaN layer has been deposited on metallic surfaces (such as tantalum alloy [30], and AISI 316L stainless steel [46]) to improve the mechanical properties and anti-corrision performance, and the hardness of TaN films could increase up to approximately 40 GPa. Therefore, it was expected that the enhanced mechanical properties of the TaN-coated Ti could have great potential to be used in synthetic bone/dental material or coatings of implants.

### 3.5. Microbiologically induced corrosion behaviors of the coated Ti

**Fig 4:**
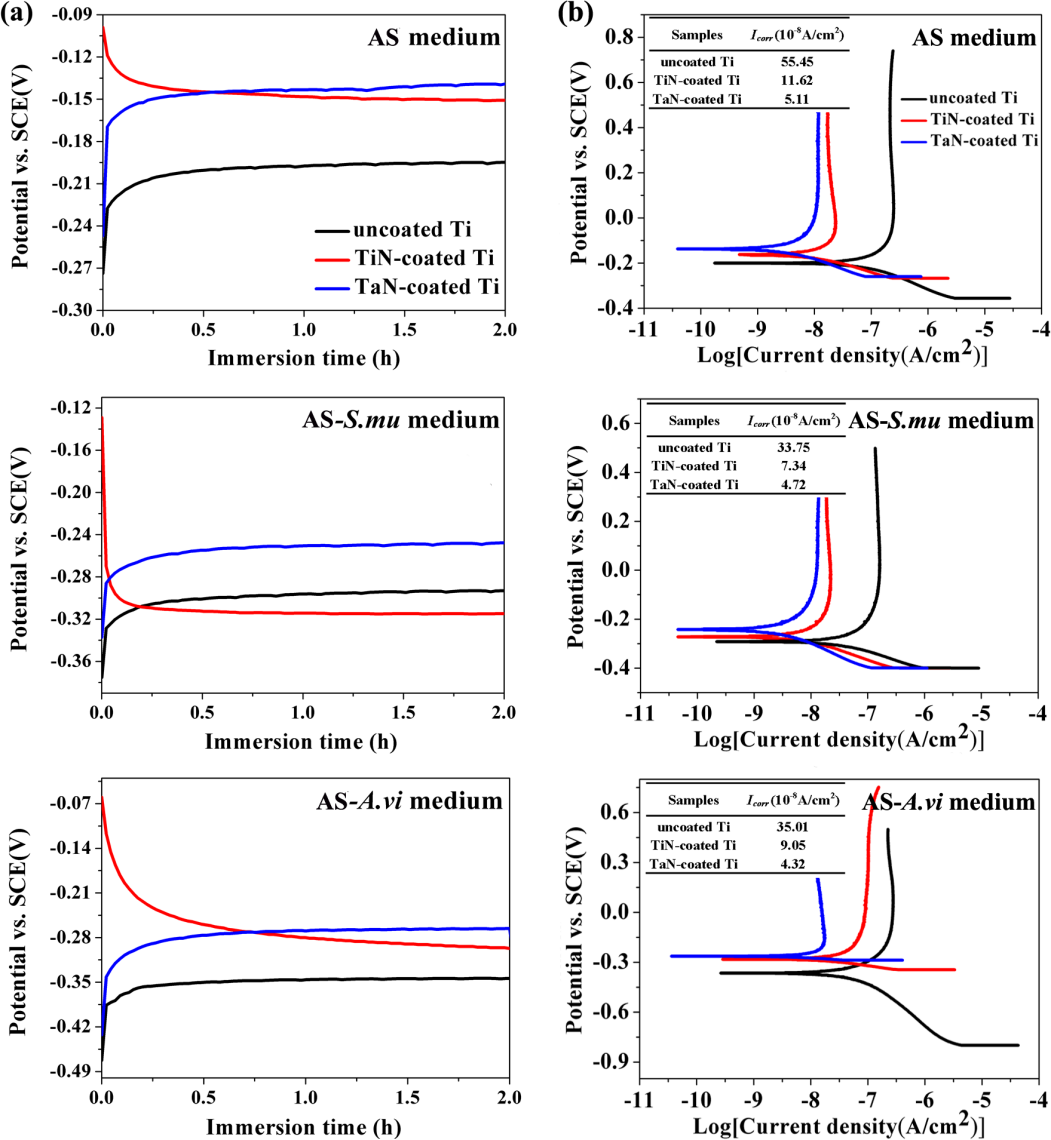
Open circuit potentials (a) and potentiodynamic polarization curves (b) of the uncoated Ti, TiN-coated Ti, and TaN-coated Ti in AS, AS-S.mu, and AS-A.vi solutions, respectively. The inset table in (b) shows the corrosion current density (*I*_*corr*_) of the samples.

MIC of metallic materials occurs by way of the main microbial metabolites such as inorganic acids, organic acids, sulfide, ammonia and others. These metabolites enhance the corrosive environment and change the characteristics of the environment around the metals, including the oxygen concentration, salinity, and acidity, thus resulting in the formation of a local oxygen concentration cell [47,48]. Previously, it is reported that Ti-based materials is vulnerable to the severe corrosion by the metabolic activity of living micro-organisms [5]. In the oral environment, dental materials have to be provided with good corrosion resistance. The bio-corrosion of dental materials can weaken their service life and the elements released from them may have detrimental effect on local tissues or accumulate elsewhere in the body. Thus, it is critical to reduce the microbial corrosion of the dental implants made of Ti-based materials. Fig 4 showed the OCP and potentiodynamic polarization curves of pure Ti and decorated Ti samples in AS, AS-*S*.*mu* and AS-*A*.*vi* soluions. *S*. *mutans*, the major pathogen responsible for dental caries [49], and *A*. *viscosus*, one of the most common pathogens associated with periodontal infection and oral abscesses [50], were selected as representatives. OCP is the potential of the working electrode relative to the reference electrode without any applied current, reflecting the thermodynamic equilibrium at the interface of metal and solution as a function of time. As seen in Fig 4(A), the OCP curves of neat Ti and TaN-coated Ti took on an upward trend and became stable gradually after 0.5 h in all three solutions. However, the OCP curves of TiN-coated Ti groups declined sharply at the initial stage and were then stable at a low potential. By comparing the three solutions, the introduction of bacteria aggravated the corrosion behaviors of all Ti samples seriously with the reduction of OCP values, especially for *A*. *viscosus*. This was possibly due to the high increase of lactic acid produced by *A*. *viscosus*, accelerating the corrosion of Ti. Notwithstanding the stabilized OCP of TiN-coated Ti was more positive than polished Ti, TiN coating seemed more vulnerable to the corrosion in the presence of *S*. *mutans*. In addition, the TaN-coated Ti had the most noble OCP level compared with bare Ti and TiN-coated Ti in three solutions, suggesting of the inert nature and superior corrosion resistence of TaN coatings. The potentiodynamic polarization curves were measured in various bacteria-containing AS solutions, as shown in Fig 4(B). Consistent with the results of OCP, the presence of bacteria caused a shift in *E*_*corr*_ to more negative values than those of AS medium, indicating that microbes reduced the passivity of samples and enhanced the dissolution rate of them. Moreover, *A*. *viscosus* involved with the lowest *E*_*corr*_ and induced more severe bio-corrosion than *S*. *mutans*. *A*. *viscosu*s influences the corrosion activity through releasing organic and inorganic metabolites in addition to oxygen and carbon dioxide consumption. In particular, *A*. *viscosus* produces lactic acid, proteinase, hydrolytic enzymes and succinate under anaerobic conditions [51,52]. Lactic acid is proved to provoke a marked attack on the Ti surface and the existence of enzymes can also enhance the corrosion of metals [47], which may explain why *A*. *viscosus* contributes to more severe corrosion behaviors. In three solutions, all the coated samples have lower corrosion current density (*I*_*corr*_) than that of bare Ti. Notably, TaN-coated Ti group displayed a much lower *I*_*corr*_ (5.11, 4.72 and 4.32×10^−8^ A/cm^2^, respectively in three solution) than others at the anode curves. These values suggested that the TiN and TaN coatings could effectively increase the anti-corrosion properties of Ti substrate in AS and bacteria-containing AS at 37°C. And the TaN film provided the most satisfactory protection upon Ti among other samples.

**Fig 5:**
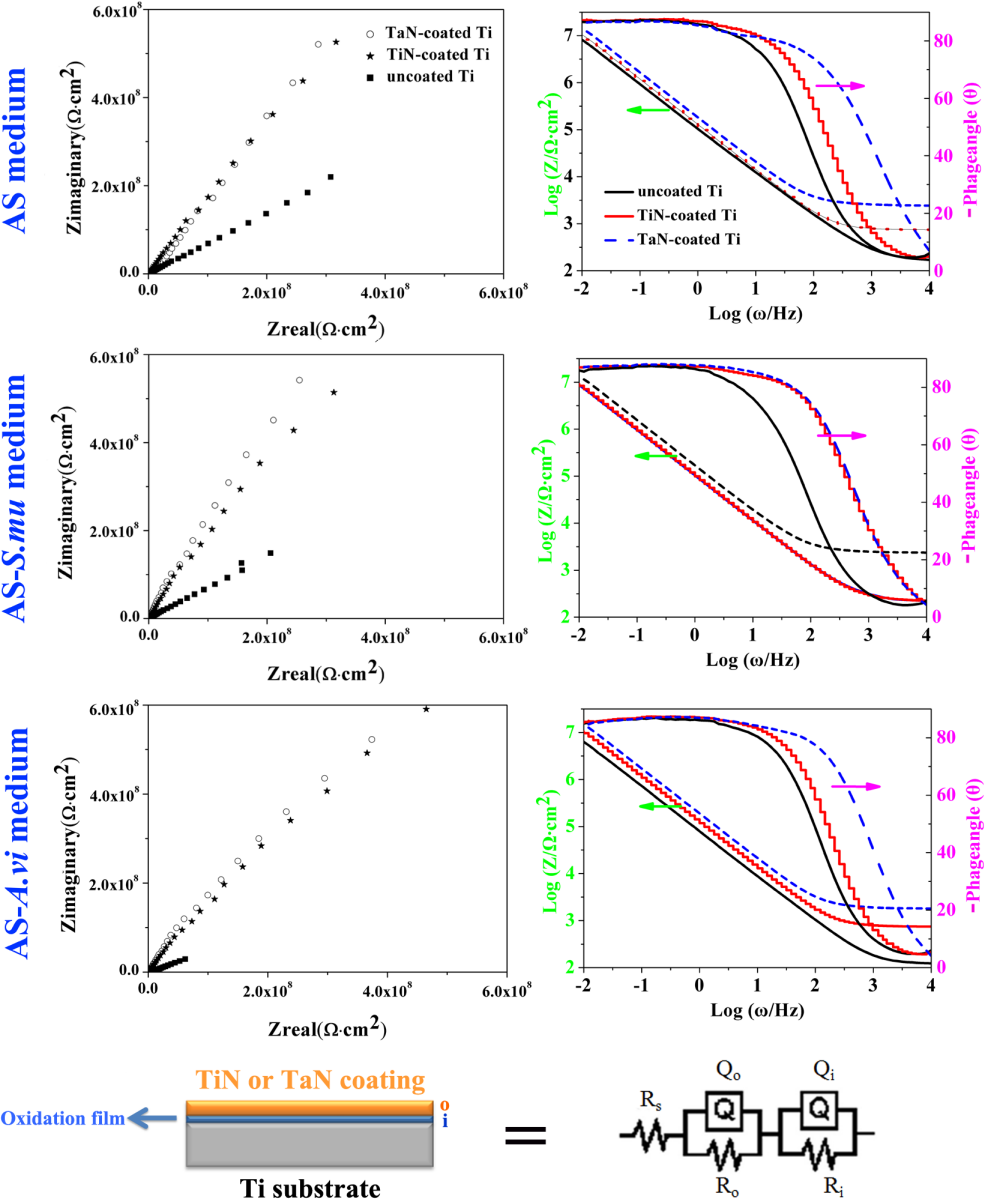
Representative EIS spectrum plots of the pristine Ti, TiN-coated Ti, and TaN-coated Ti in AS, AS-*S*.*mu*, and AS-*A*.*vi* solutions: Nyquist plots (a), Bode plots (b), and the equivalent electrical circuit model (c).

In order to furter study the stability and corrosion resistance of the TiN/TaN coatings, electrochemical impedance spectroscopy (EIS) of the samples at *E*_*corr*_ after immersion 2 h in AS and baterial-containing AS media were presented, respectively, in the form of Nyquist plots in Fig 5(A) and Bode-phase and Bode-magnitude plots in Fig 5(B). It could be seen from the Nyquist plots that the diameters of the semicircle for the coated samples were bigger in comparison with that of pristine Ti in all three solutions, which suggested the increase of the corrosion resistance of the samples, with sort order of TaN-coated > TiN-coated > Ti. As shown in Fig 5(B), there were two distinct regions for all experimental materials characterized in Bode plots. In the high frequency range (10^3^−10^4^ Hz), a flat portion of curves (slope≈ 0) was observed in the Bode-magnitude plots, while the phase angle drops to 0° in the Bode-phase angle plots, which was due to the response of electrolyte resistance. In the broad low and middle frequency ranges, the spectra displayed a linear slope of about -1 in the Bode-magnitude plots, and the phase angle values approximate -85° in AS and bacteria-containing AS. This indicated a typical passive film presented on the surface and a near capacitive response for passive film characterized in three AS solutions. EIS is a tool used to analyze complex electrochemical systems through evaluation with equivalent circuit modeling. In the present study, the R_s_(Q_i_R_i_)(Q_o_R_o_) equivalent circuit model based on a two layer structure (an inner compact layer and an outer relatively porous layer) was proposed to fit the EIS data for coated Ti substrate, and R_s_(Q_i_R_i_) model for the uncoated Ti. The R_s_(Q_o_R_o_)(Q_i_R_i_) equivalent circuit was shown in Fig 5(C) in which R_s_ represented the solution resistance of the electrolyte, R_o_ represented the resistance of the outer porous coatings, R_i_ represented the resistance of the compact passive layer, Q_o_ represented the capacitance of the outer porous coatings, and Q_i_ represented the capacitance of the compact passive layer. The parameter values obtained by the fitting procedure were shown in Table C in S1 File, and the circuit displayed a perfect fit for the experimental data with chi-square (**χ**) values below 10^−3^. As seen in Table C in S1 File, it was clear that the passive film formed on pristine Ti became defective or unstable after immersion in media containing bacteria supported by the lower R_i_ and higher Q_i_ values, combined with smaller diameters of the semicircle in Nyquist plots, especially for *A*. *viscosus*-containing ones, consistent with OCP and Tafel plots results. As we known, Ti and its alloys have a low corrosion resistance in oral circumstance because the formed passive film on their surfaces could be slowly attacked due to the byproducts (such as extracellular polysaccharides, organic acids) secreted by oral microorganisms, hence, inactive coatings should be encouraged to greatly increase the bio-corrosion resistance of Ti-based implants. In comparison to pristine Ti, the coated samples presented higher R_o_, and the TaN-coated counterparts possessed the highest R_o_ values than other groups in three AS solutions, which predicted its best improvement in the corrosion resistance of Ti. In terms of the TaN-decorated Ti, the TaN layer provided effective and obvious corrosion protection on the substrate through acting as an insulating barrier. Meanwhile, the uniform/compact coating morphology, and the strong binding strength of the TaN coatings to Ti substrate (26.7 N) co-contributed to preventing penetrating of bacteria-containing solution into the coatings and protecting the Ti substrate from MIC.

### 3.6. Evaluation of biofilm formation

**Fig 6:**
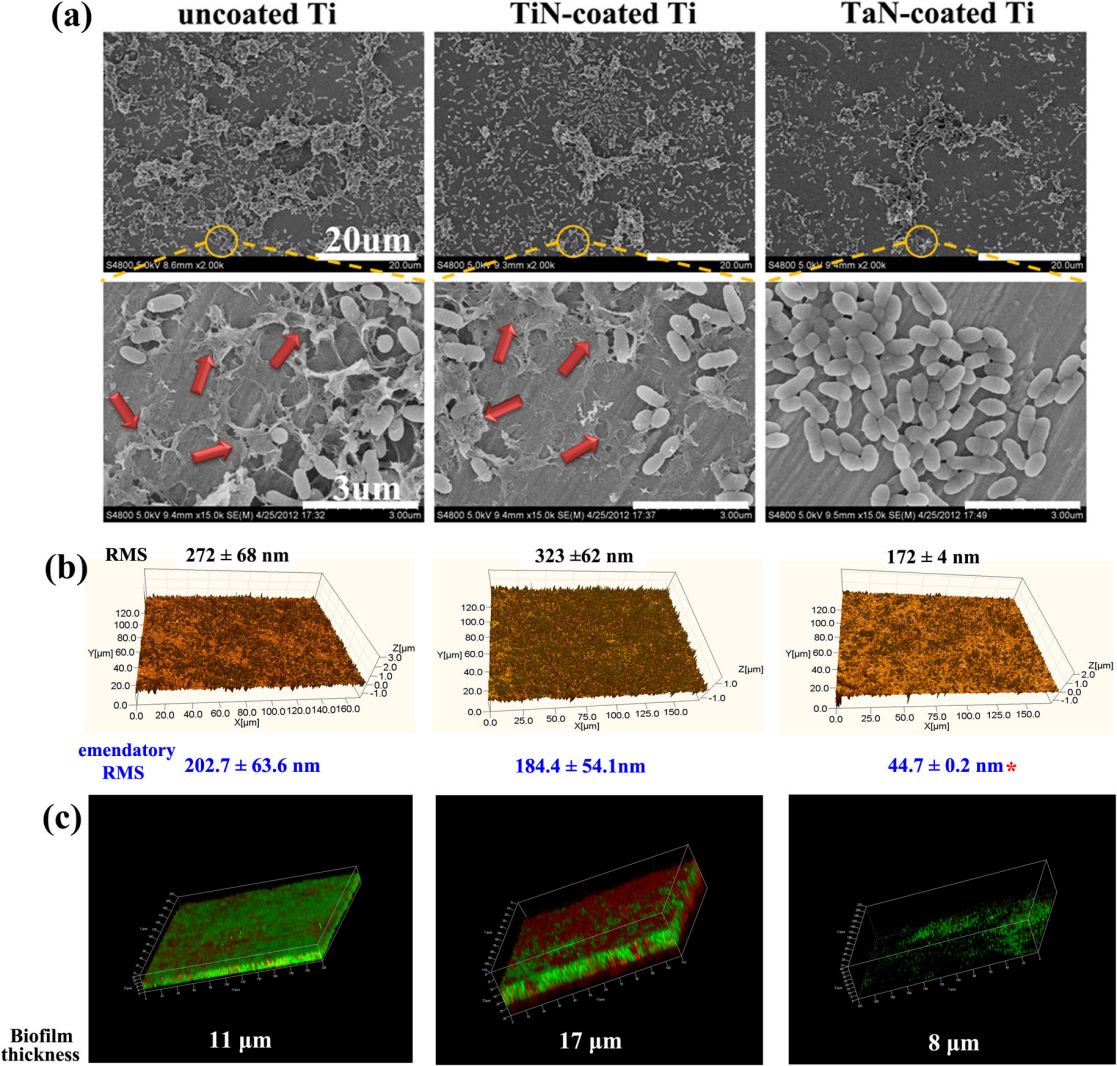
Biofilm formation observation of the pristine Ti, TiN-coated Ti, and TaN-coated Ti after 14 days of incubation with mixed bacteria: SEM images of bacteria and EPS (a), WLI images (b), and Live/dead cell staining of CLSM. In WLI images, the brown represents bacterial cells, and the orange represents substrates. Red arrows in (a) point to the secreted EPS of bacteria. * represents *p* < 0.05 compared with other groups.

In the oral cavity, planktonic bacteria first adhere to an implant interface and ultimately evolve into biofilm. The biofilm refers to the association of bacteria into multicellular consortia, consisting of still autonomous cells connected by an extracellular matrix, which defend against harmful environmental physical and chemical factors, and lead to localized corrosion and deterioration of the substratum materials [53,54]. Multiple corrosion-causing bacteria may synergistically lead to much more severe corrosion of the attached metals than one single strain [55]. Actually, there are a wide variety of commensal bacteria in supragingival oral environment. Thus, the coordinated effects of three common bacteria including *S*. *mutans*, *A*.*viscosus*, and *P*. *gingivalis* on the coated samples were probed in this study. As illustrated in Fig 6(A), a great number of mixed bacterial cells were found on the pristine Ti and TiN-decorated Ti surfaces after 14 days culture. From the enlarged images, it could be seen that biofilm formed on both pure Ti and TiN-coated Ti surfaces consisted of bacteria and the secreted extracellular polymeric substances (EPS). EPS produced by biofilms could not only act as a contributor to enhance the bio-corrosion of metallic materials, but also make bacteria inside very difficult to eradicate. Compared with other groups, however, less bacterial cells without EPS were observed on the TaN-decorated Ti, suggesting TaN coating had a negative influence on the formation of biofilm.

WLI is a powerful and well-established technique for noncontact measurement of biological samples under physiological conditions, and it requires minimum sample preparation. It can quickly determine three-dimensional (3-D) surface topography and roughness by means of a non-destructive methodology over larger areas at high vertical and moderate lateral resolution. Fig 6(B) showed the 3-D interference profile of the bacteria accumulation on the coupon surfaces. There was the maximum of mixed bacteria on TiN-coated Ti sample, implying TiN coating was prone to bacterial proliferation. In accordance with SEM analysis, fewer bacteria clustered on the TaN-coated Ti than pure Ti and TiN-coated Ti. Furthermore, the roughness of biofilms was analyzed quantitatively. Albeit the surface morphology of the TiN-coated and TaN-coated Ti was different, after subtracting the coating surface RMS effect in Fig 2, the biofilm formed on TaN-coated Ti surfaces (44.7 ± 0.2 nm) was significantly reduced than that on bare Ti and TiN-coated Ti (*p*< 0.05) at the 14^th^ day, which suggested that TaN coating exhibited a certain degree of antibacterial property. Fig 6(C) showed CLSM images of 14 day bacterial biofilms with the LIVE/DEAD BacLight bacterial viability kit. For the pristine Ti surface, extensive bacterial biofilm was formed with visible dead and live bacteria, and the biofilm on TiN-coated Ti reached about 17 μm thickness. In sharp contrast, the fewest amount of bacterial cells (≈ 8 μm) were detected on TaN-coated Ti group, in agreement with SEM and WLI observations, further suggesting that the antimicrobial behavior of TaN-coated Ti implant could be preserved for an extended period of time (14 days). Although, previous studies show that TaO or TaN coating has obvious antimicrobial capability towards *Staphylococcus aureus* and *Actinobacillus actinomycetemcomitans* probably because of coating structures (amorphous and crystalline phases) [31,32], the real reason that TaN addition improves the antimicrobial performance of Ti-based materials remains not very clear. Therefore, an overall investigation shall be conducted on the antibacterial mechanism of TaN coating in the future.

### 3.7. Surface microstructure of the bio-corrosive coated Ti

**Fig 7:**
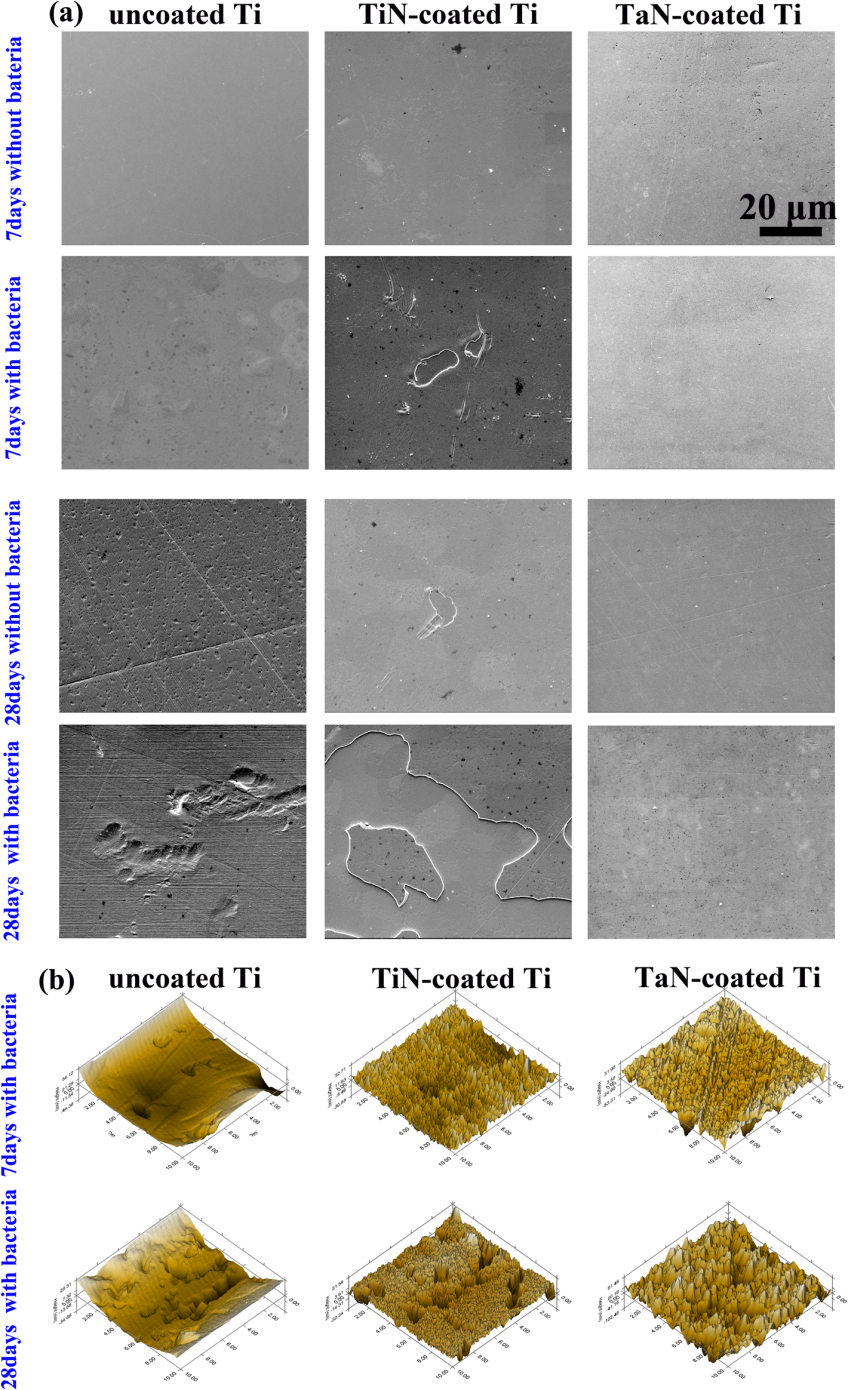
The SEM micrographs (a) and *in situ* SPM (b) of the pure Ti, TiN-coated and TaN-coated surfaces exposed to medium with and without the mixed bacteria for 7 and 28 days after the removal of biofilm and corrosion products.

The deleterious impact of bacterial colonization on the tested surfaces was assessed by profiling the microstructures on the coupon surfaces after removal of the biofilms and deposits for 7 and 28 days as shown in Fig 7. After the 7 days of immersion in medium with bacteria, the breakdown of TiN coating was seen in TiN-coated Ti, though there was no visible alteration on surfaces for three Ti samples in medium without bacteria for 7 days. Over the subsequent period (28 days), extensive localized corrosive micropits occurred on pure Ti and evident detachment of TiN layer after incubation in medium without bacteria was observed. It was obvious that the presence of bacteria seriously accelerated the MIC of Ti and TiN-coated Ti samples, confirmed by the aggravated corrosion pit of Ti and severe detachment of TiN coating in Fig 7(A). Bacteria have been reported to have the capacity to produce a pitting corrosion phenomenon on exposed titanium surfaces, leading to significant deterioration in the mechanical properties of the implant [48]. The destruction of TiN coating makes it easier for corrosive liquid to diffuse into the coating and renders the Ti substrate susceptible to corrosion. However, TaN-coated Ti coupons incubated in either the bacteria-free or the bacteria-containing medium always exhibited intact, pit-free and well-anchored surfaces on the 7^th^ and 28^th^ days, demonstrating its excellent bio-corrosion resistance to bacteria, and the strong binding strength of the TaN coatings to Ti substrate. The same phenomenon was found in *in situ* SPM images (Fig 7(B)) immersed in media containing bacteria for 28 days.

**Fig 8:**
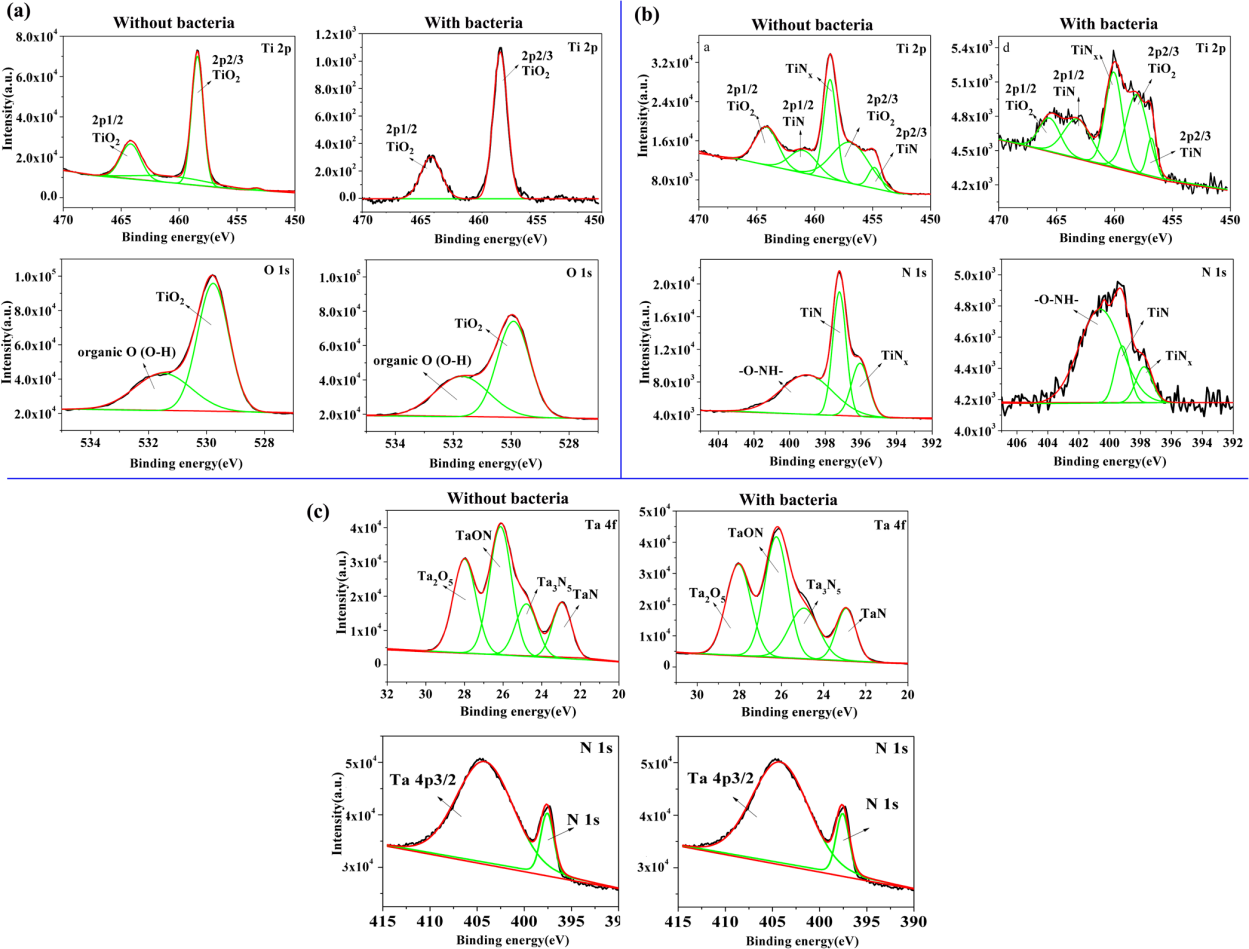
The XPS high-resolution spectra of Ti 2p and O 1s for bare Ti (a), Ti 2p and N 1s for TiN-coated Ti (b), and Ta 4f and N 1s for TaN-coated Ti samples (c) in medium with and without the mixed bacteria for 28 days after the removal of biofilm and corrosion products.

To better understand the changes in the chemical composition induced by the biofilm, XPS measurement was carried out to obtain the elemental information of the outermost layer on the coupons after the biofilm removal. As shown in Figure B in S1 File and Table D in S1 File, Ti and O peaks for pure Ti after immersion in bacteria-containing medium were reduced than that in medium without bacteria for 28 days. With regard to TiN coatings, the most noticeable change was the decrease of Ti and N contents in medium with bacteria, resulting from the corrosion of the layer and the dissolution of TiN. Furthermore, based on XPS high-resolution spectrum of bare Ti surface (Fig 8(A)), the intensity of the TiO_2_ peaks in Ti 2p and O 1s decreased dramatically after bio-corrosion experiment. Metal and its alloys for dental applications are known to rely on their surface oxides for corrosion resistance in the oral environment. The above EIS results of corrosion tests showed that pure Ti exhibited a statistically lower R_i_ value after immersion in media containing microorganism (Table C in S1 File). It could be concluded that the reduction in the relative surface levels of O and Ti resulted in a decrease in corrosion resistance (R_i_). These data confirmed that the oxide layer formed on the outermost Ti surface (TiO_2_) was impaired under 28 days of immersion in bacterial medium on account of a lower pH promoted by the release of acidic substances from microbes. Three main differences were distinguished for TiN-coated Ti after 28 days of exposure in bacteria-containing medium: 1) Ti 2p and N 1s spectra become more fluctuated; 2) the signals of TiN peaks were weakened in the Ti 2p and N 1s spectra; 3) the amount of organic N (-O-NH-) was increased in N 1s spectra. The changes could be ascribed to the detachment of TiN coating. *S*. *mutans*, *A*. *viscosus* and *P*. *gingivalis* can metabolize carbohydrates to lactic acid, [56] which is one of the byproducts. The production of acid may create corrosive conditions on the Ti and TiN-coated Ti surface. The glucosyltransferase and glucan binding protein on the bacterial cell surface also affect the corrosion behavior of the samples [57,58]. Our results showcased that byproducts of mixed bacterial metabolism participated in breaking down the oxide layer and TiN in the experimental groups, resulting in altered samples corrosion behaviors. However, in accordance with XPS wide scan result of TaN-coated Ti in S2(c) Fig, the high-resolution spectra of Ta 4f and N 1s also remained constant throughout the exposure period, indicating that TaN film was stable in the bacterial medium and possessed adhesive strength to Ti substrate. As a consequence, compared with pure Ti and TiN coating, our results have demonstrated that TaN coating provided the most satisfactory protection and better antibacterial property, as well as the enhanced mechanical property upon Ti.

## Conclusion

In summary, the novel TaN-coated Ti material was successfully prepared on Ti sheet using magnetron sputtering approach, and the microbial corrosion behaviors of the TaN-coated Ti surface were evaluated for the first time. Mechanical test indicated that TaN layer was benefit to increase the hardness and modulus of the neat Ti. After 14 days incubation, TaN-decorated Ti induced good *in vitro* antibacterial activity toward the mixed bacteria including *S*. *mutans*, *A*. *viscosus* and *P*. *gingivalis*. More importantly, the TaN coating significantly enhanced the the resistance of MIC, strong binding strength, and provided the most satisfactory protection to Ti in both AS and bacteria-containing AS solutions than pure Ti and TiN coating. A further comprehensive *in vivo* bio-corrosion evaluation of the TaN-coated Ti is currently underway in our laboratory. In comparison to other reported oxide, nitride, and carbide thin films deposited on Ti surface, such as ZrO_2_, IrO_2_, SiC, and TiCN, which only improves the mechanical strengh and corrosion resistance for Ti in bacterial-free solutions, the advantage of the TaN coating is that it could endow dental implant with promoted resistance to MIC, and antibacterial behavior combined improvement in mechanical property, simultaneously. Besides, our results demanstrated that oral bacteria had a strong influence on the bio-corrosion activity of dental implant surface, so that microbial corrosion should be considered one of the key criteria for assessing the biocompatibility of dental implant materials. The study could pave the way for the fabrication and employment of TaN to be used as dental implant or coating material for dental applications.

## Supporting Information

S1 File Figure A. The XPS high-resolution N 1s spectra of TiN-coated (a) and TaN-coated Ti (c), as well as the high-resolution O 1s spectra for TiN-coated (b) and TaN-coated Ti (d). Figure B. The XPS wide scan spectra of the bare Ti (a), TiN-coated Ti (b), and TaN-coated Ti in medium with and without the mixed bacteria for 28 days after the removal of biofilm. Table A. Elemental composition of the pristine Ti, TiN-coated and TaN-coated Ti determined by XPS analysis. Table B. The contact angle and surface energy of the pristine Ti, TiN-coated and TaN-coated Ti samples. Table C. The fitted electrochemical parameters of the pristine Ti, TiN-coated Ti, and TaN-coated Ti in AS, AS-*S*.*mu*, and AS-*A*.*vi* solutions, respectively. Table D. The relative contents of main elements (Ti, Ta, O and N) of the pristine Ti, TiN-coated and TaN-coated Ti determined by XPS analysis in medium with and without the mixed bacteria for 28 days after the removal of biofilm.
(DOCX)
