# Supplementary material for: Tantalum Nitride-Decorated Titanium with Enhanced Resistance to Microbiologically Induced Corrosion and Mechanical Property for Dental Application
Source: PLoS One. 2015 Jun 24;10(6):e0130774. doi: 10.1371/journal.pone.0130774 (PMC4479376; doi:10.1371/journal.pone.0130774)
Supplement: S1 File — The XPS high-resolution N 1s spectra of TiN-coated (a) and TaN-coated Ti (c), as well as the high-resolution O 1s spectra for TiN-coated (b) and TaN-coated Ti (d). Figure B. The XPS wide scan spectra of the bare Ti (a), TiN-coated Ti (b), and TaN-coated Ti in medium with and without the mixed bacteria for 28 days after the removal of biofilm. Table A. Elemental composition of the pristine Ti, TiN-coated and TaN-coated Ti determined by XPS analysis. Table B. The contact angle and surface energy of the pristine Ti, TiN-coated and TaN-coated Ti samples. Table C. The fitted electrochemical parameters of the pristine Ti, TiN-coated Ti, and TaN-coated Ti in AS, AS-S.mu, and AS-A.vi solutions, respectively. Table D. The relative contents of main elements (Ti, Ta, O and N) of the pristine Ti, TiN-coated and TaN-coated Ti determined by XPS analysis in medium with and without the mixed bacteria for 28 days after the removal of biofilm. (DOCX) [file pone.0130774.s001.docx]

**Combined Supporting Information S1 File**

**Figure A. The XPS high-resolution N 1s spectra of TiN-coated (a) and TaN-coated Ti (c), as well as the high-resolution O 1s spectra for TiN-coated (b) and TaN-coated Ti (d).**


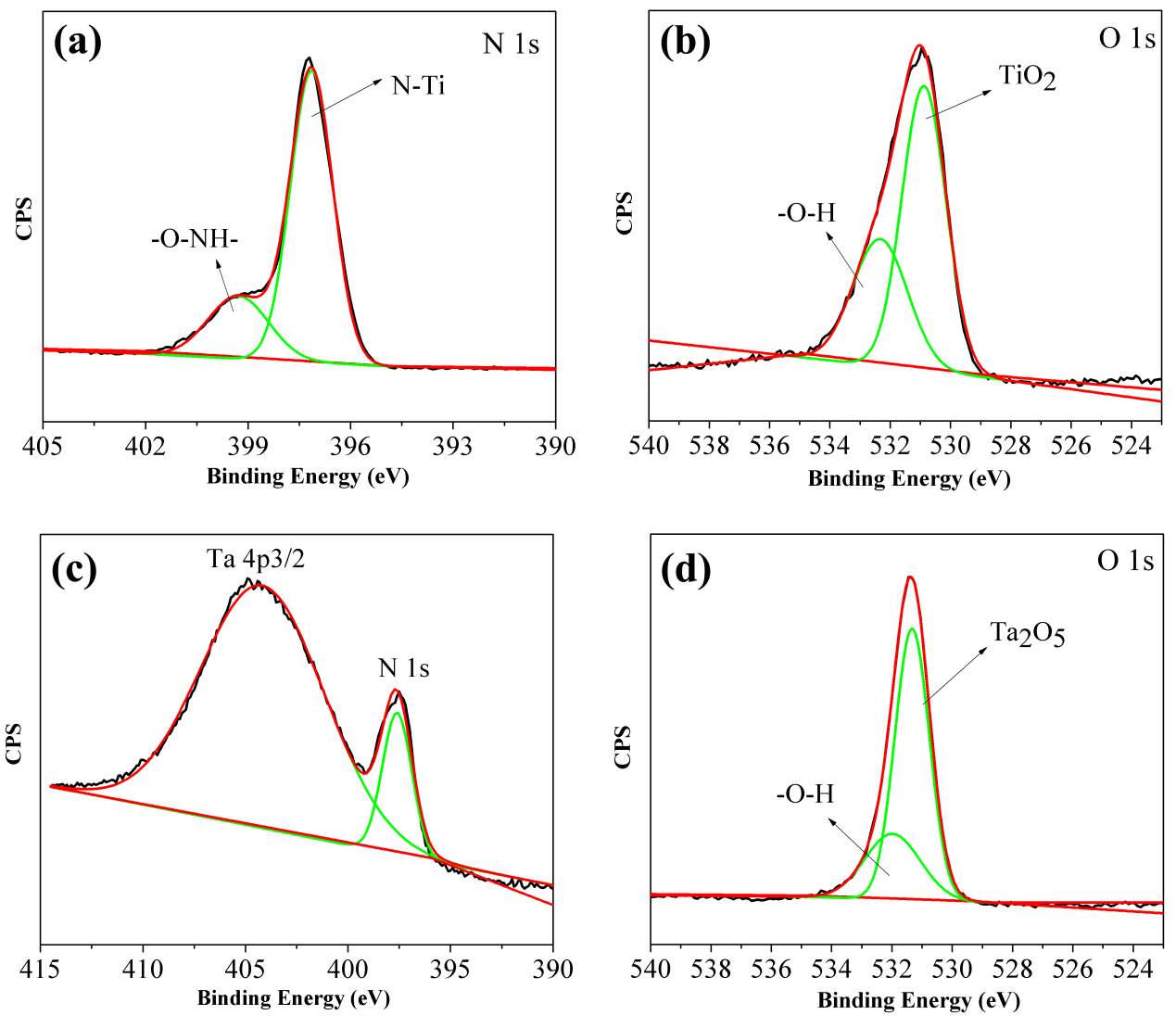


**Figure B. The XPS wide scan spectra of the bare Ti (a), TiN-coated Ti (b), and TaN-coated Ti** **in medium with and without the mixed bacteria for 28 days after the removal of biofilm.**

**
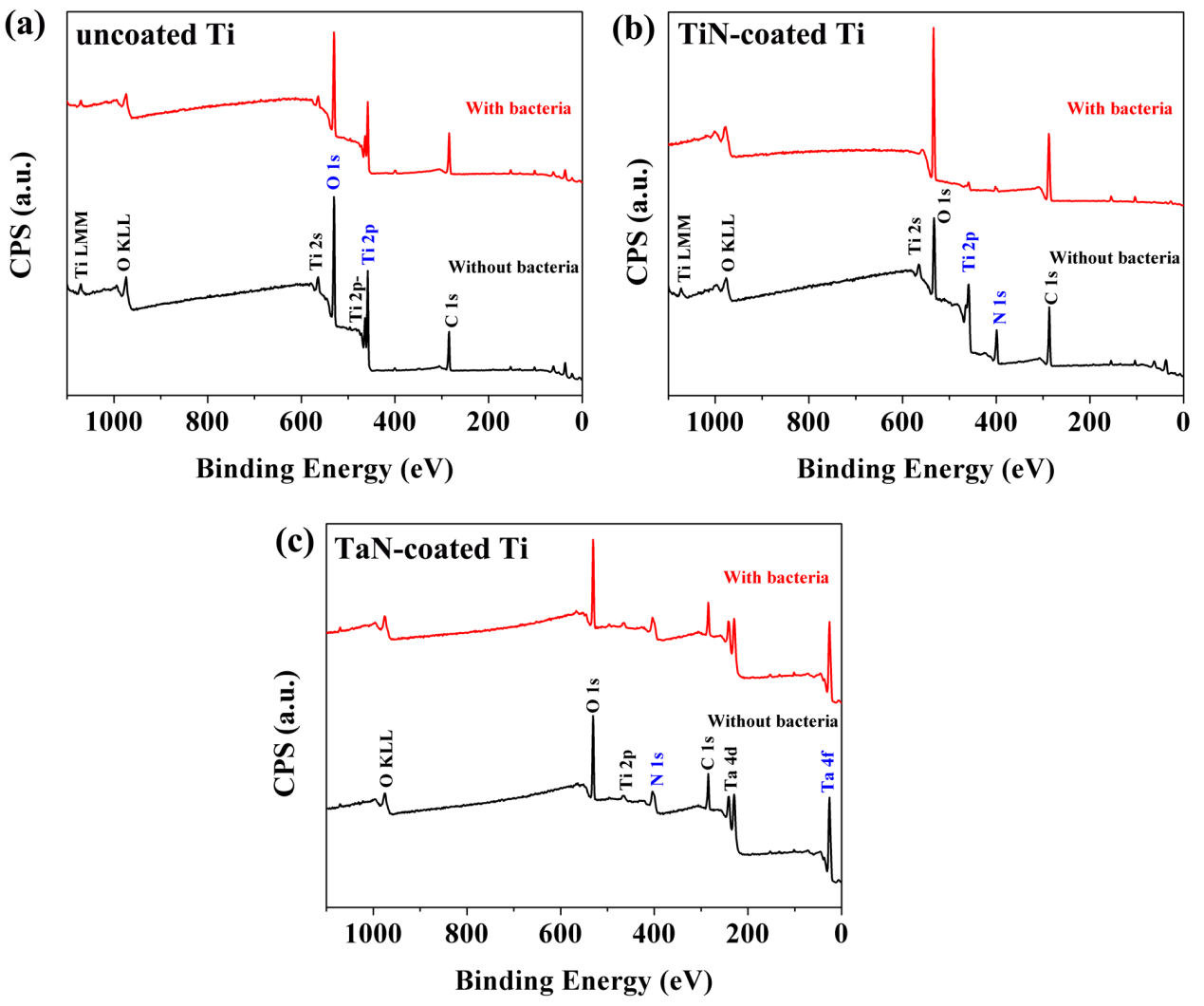
**

**Table A. Elemental composition of the pristine Ti, TiN-coated and TaN-coated Ti determined by XPS analysis.**

| [**Element**](app:ds:element)**s** | **uncoated Ti** | **TiN-coated Ti** | **TaN-coated Ti** |
| --- | --- | --- | --- |
| **Ti** | 52.27 % | 41.35 % | 16.56 % |
| **C** | 9.69 % | 9.27 % | 3.91 % |
| **O** | 38.04 % | 30.11 % | 33.90 % |
| **N** | 0 % | 19.27 % | 14.09 % |
| **Ta** | 0 % | 0 % | 31.54 % |

**Table B. The contact angle and surface energy of the pristine Ti, TiN-coated and TaN-coated Ti samples.**

| **Samples** | **uncoated Ti** | **TiN-coated Ti** | **TaN-coated Ti** |
| --- | --- | --- | --- |
| **Contact angle (°)** | 75.1 ± 2.6 | 76.2 ± 2.1 | 76.3 ± 0.9 |
| **Surface energy (J/m)** | 40.8 ± 2.7 | 41.9 ± 1.7 | 41.4 ± 0.8 |

**Table C. The fitted electrochemical parameters of the pristine Ti, TiN-coated Ti, and TaN-coated Ti in AS, AS-*S.mu*, and AS-*A.vi* solutions, respectively.**

| **Conditions** | **Samples** | **R_s_ (Ω·cm^2^)** | **Q_i_ (μF·cm^-2^)** | | **R_i_ (kΩ·cm^2^)** | | **Q_o_ (μF·cm^-2^)** | | **R_o_ (kΩ·cm^2^)** | | **χ** |
| --- | --- | --- | --- | --- | --- | --- | --- | --- | --- | --- | --- |
| **AS medium** | uncoated Ti | 88.4 ± 4.8 | 46.3 ± 5.4 | 9.16 ± 1.57 | | / | | / | | < 10^-3^ | |
|  | TiN-coated Ti | 87.3 ± 7.2 | 14.5 ± 1.4 | 8.31 ± 2.15 | | 28.5 ± 2.8 | | 12.2 ± 3.7 | | < 10^-3^ | |
|  | TaN-coated Ti | 79.5 ± 3.4 | 16.7 ± 2.7 | 9.87 ± 0.26 | | 11.3 ± 3.7 | | 44.6 ± 3.3 | | < 10^-3^ | |
| **AS-*S.mu* medium** | uncoated Ti | 89.6 ± 8.8 | 55.3 ± 6.4 | 5.81 ± 0.24 | | / | | **/** | | < 10^-3^ | |
|  | TiN-coated Ti | 76.9 ± 6.4 | 9.87 ± 2.6 | 4.81 ± 1.35 | | 16.1 ± 1.9 | | 4.61 ± 0.65 | | < 10^-3^ | |
|  | TaN-coated Ti | 74.2 ± 7.1 | 10.3 ± 4.1 | 4.47 ± 1.69 | | 14.5 ± 2.2 | | 21.1 ± 7.6 | | < 10^-3^ | |
| **AS-*A.vi* medium** | uncoated Ti | 46.9 ± 4.6 | 79.2 ± 4.9 | 1.52 ± 0.13 | | / | | **/** | | < 10^-3^ | |
|  | TiN-coated Ti | 50.6 ± 6.6 | 9.11 ± 0.76 | 2.63 ± 0.84 | | 8.14 ± 1.16 | | 2.11 ± 0.13 | | < 10^-3^ | |
|  | TaN-coated Ti | 52.7 ± 3.5 | 5.26 ± 0.37 | 2.37 ± 1.14 | | 3.21 ± 0.63 | | 14.7 ± 2.5 | | < 10^-3^ | |

**Table D. The relative contents of main elements (Ti, Ta, O and N) of the pristine Ti, TiN-coated and TaN-coated Ti determined by XPS analysis in medium with and without the mixed bacteria for 28 days after the removal of biofilm.**

| **Samples** | **Ti (%)** | **Ta (%)** | **O (%)** | **N (%)** |
| --- | --- | --- | --- | --- |
| **uncoated Ti without bacteria** | 49.61 | 0 | 34.95 | 0.38 |
| **uncoated Ti with bacteria** | 22.13 | 0 | 16.84 | 0.45 |
| **TiN-coated Ti without bacteria** | 36.51 | 0 | 20.36 | 14.10 |
| **TiN-coated Ti with bacteria** | 9.12 | 0 | 45.28 | 2.52 |
| **TaN-coated Ti without bacteria** | 9.89 | 38.61 | 26.65 | 18.18 |
| **TaN-coated Ti with bacteria** | 10.03 | 37.92 | 26.90 | 17.62 |
